# Associations between insulin resistance and low back pain risk in US adults: a cross-sectional study

**DOI:** 10.3389/fmed.2025.1538754

**Published:** 2025-04-28

**Authors:** Zhiqiang Que, Dingqiang Chen, Huirong Cai, Weibin Lan, Yuxuan Huang, Gang Rui

**Affiliations:** ^1^Department of Orthopedics, The First Affiliated Hospital of Xiamen University, School of Medicine, Xiamen University, Xiamen, China; ^2^The School of Clinical Medicine, Fujian Medical University, Fuzhou, China; ^3^Department of Orthopedics, Longyan First Affiliated Hospital of Fujian Medical University, Longyan, China; ^4^Xiamen Key Laboratory of Clinical Efficacy and Evidence Studies of Traditional Chinese Medicine, The First Affiliated Hospital of Xiamen University, School of Medicine, Xiamen University, Xiamen, China

**Keywords:** insulin resistance, National Health and Nutrition Examination Survey, triglyceride-glucose index, cross sectional study, low back pain

## Abstract

**Background:**

Insulin resistance is one of the major pathophysiological features of type 2 diabetes mellitus. Studies have revealed the association between type 2 diabetes mellitus and low back pain. However, few studies explored the relationship between insulin resistance and low back pain directly. Therefore, this study selected HOMA-IR, TyG, TyG-BMI, TyG-WC, and TyG-WtHR as indicators of insulin resistance to comprehensively investigate the association between insulin resistance and low back pain.

**Methods:**

The data for this cross-sectional study were from NHANES. Multivariate logistic regression was used to assess the association of insulin resistance with low back pain, and the stability of the results was evaluated by stratified analysis.

**Results:**

A total of 6,126 adult participants were included in the study, including 3,657 non-LBP participants and 2,469 LBP patients. All of these five indices showed significant association with low back pain after full adjustment for all covariates (Model 3), HOMA-IR [OR = 1.052, 95% CI (1.018, 1.087), *p* = 0.003], TyG [OR = 1.431, 95% CI (1.013, 2.021), *p* = 0.042], TyG-BMI [OR = 1.003, 95% CI (1.002, 1.005), *p* < 0.0001], TyG-WC [OR = 1.001, 95% CI (1.001, 1.002), *p* < 0.0001], TyG-WtHR [OR = 1.268, 95% CI (1.155, 1.393), *p* < 0.0001]. The relationship between insulin resistance and low back pain is stable in most stratified populations (*p*-interaction >0.05).

**Conclusion:**

Insulin resistance is associated with an increased risk of low back pain. The HOMA-IR, TyG, TyG-WC, TyG-BMI, and TyG-WtHR all showed a stable correlation with low back pain. TyG-BMI, TyG-WC, and TyG-WtHR are more stable in their associations with low back pain than TyG alone.

## 1 Introduction

Low back pain (LBP) is characterized by pain, muscle tension, or stiffness located between the costal margin and the subgluteal folds, and may be accompanied by leg pain (sciatica) and neurological symptoms in the lower limbs (1–3). LBP can be classified as acute (lasting up to 6 weeks), subacute (lasting 6 weeks to 3 months), or chronic (lasting 3 months or longer) (1). Based on etiology, LBP is categorized as either specific or non-specific. Specific LBP arises from identifiable pathophysiological mechanisms, such as herniated nucleus pulposus, infection, osteoporosis, rheumatoid arthritis, fractures, or tumors. Non-specific LBP is defined as LBP without a clear underlying cause (3). It is the leading cause of disability worldwide, and most people experience LBP at least once in their lifetime. The prevalence of LBP increases with age, with 1–6% in children aged 7–10 years, 18% in adolescents, and a peak prevalence of 28–42% in those aged 40 to 69 years (3–5). In western countries, the reported lifetime prevalence of LBP ranges from 49% to 70%, and the point prevalence of LBP from 12% to 30% (3). In 2015, LBP caused about 60.1 million person-years lived with disability, up 54% from 1990 (6), greatly increasing the cost of health care and social support systems. As the population ages, the prevalence of LBP is expected to markedly rise in the upcoming decades. Therefore, it is significant for the early recognition and timely intervention of LBP.

Insulin resistance (IR) refers to a disease in which the effect of insulin on tissues is weakened due to various reasons, and it cannot effectively promote the absorption of glucose by surrounding tissues and inhibit the output of glucose from the liver, increasing blood sugar (7). IR is the core pathophysiological mechanism of metabolic syndrome (MetS) (8). Researches (9, 10) have suggested that MetS may play an important role in pain. In addition, IR is one of the major pathophysiological features of type 2 diabetes mellitus (T2DM) (11). Studies (12, 13) have shown the association between T2DM and LBP, with LBP being more common in people with T2DM. Several common risk factors have also been found in T2DM and LBP, such as obesity (14, 15) and low-grade systemic inflammation (16, 17). Based on these previous researches, we speculate that there is positive association between IR and LBP. However, few studies to explored the association between IR and LBP directly. Therefore, this cross-sectional study was conducted to investigate the association between them, and comprehensively evaluated the association between various IR indices (HOMA-IR, TyG, TyG-BMI, TyG-WC, and TyG-WtHR) and LBP, to promote the early identification and scientific management of LBP.

## 2 Methods

### 2.1 Data sources

National Health and Nutrition Examination Survey (NHANES) is a study conducted by the Centers for Disease Control and Prevention (CDC) to assess the health and nutritional status of the US population.^1^ NHANES gathers data through a combination of questionnaires, physical assessments, and laboratory analyses on representative samples to comprehensively assess and track the health status and nutritional habits of the US population. These data have important implications for studying the epidemiology of disease, developing public health policies, and guiding clinical practice. This study obtained data from NHANES (1999–2000, 2001–2002, 2003–2004, and 2009–2010), a total of 41,663 participants. The National Center for Health Statistics Research Ethics Review Board approved the NHANES, and all study participants provided written informed consent.

### 2.2 Assessment of LBP

The miscellaneous pain section of NHANES (1999–2000, 2001–2002, 2003–2004, and 2009–2010) provides personal interview data on LBP. In the three cycles of 1999–2000, 2001–2002, and 2003–2004, participants were asked, “During the past 3 months, did you have LBP?” In the 2009–2010 cycle, participants were asked, “Was there one time when you had pain, aching, or stiffness in your low back on almost every day for 3 or more months in a row?” The answer “yes” indicates the presence of LBP and the answer “no” indicates non-LBP.

### 2.3 Definition of IR surrogates

We extracted fasting triglyceride, fasting glucose, fasting insulin, waist circumference (WC), height, and weight from NHANES. The calculation formulas for IR surrogates are as follows. HOMA-IR = fasting insulin (μU/mL) × fasting glucose (mmol/L)/22.5 (18). TyG index = Ln [fasting triglycerides (mg/dL) × fasting glucose (mg/dL)/2] (19), TyG-BMI index = TyG × BMI (20), TyG-WC index = TyG × WC (cm) (20), TyG-WtHR index = TyG × WC (cm)/height (cm) (20).

### 2.4 Covariates

Potential covariates were identified based on the literature and clinical experience. This study selected age, sex, ethnicity, marital status, poverty income ratio (PIR), educational level, hypertension, smoking status, alcohol use, fasting total cholesterol (mg/dL), high density lipoprotein (HDL) cholesterol (mg/dL) and low density lipoprotein (LDL) cholesterol (mg/dL) as covariates. Ethnicity was classified into four groups: “non-Hispanic White,” “non-Hispanic Black,” “Mexican American,” and “other.” Marital status was classified into “married or living with a partner” and “other.” Poverty income ratio (PIR), a ratio of family income to poverty, was classified into “0–1.3 PIR,” “>1.3–3.5 PIR,” “>3.5 PIR.” Education level was classified into “Less Than 9th Grade,” “High School Grade or Equivalent” and “College Graduate or above.”

### 2.5 Study participants

Participants for this study were drawn from the NHANES (1999–2000, 2001–2002, 2003–2004, and 2009–2010). Exclusion criteria: (1) missing LBP data; (2) missing data on IR indices; (3) missing weight data or having a weight value of zero.

### 2.6 Statistical analysis

All data processing and statistical analysis were performed using the R software (version 4.3.2). For continuous variables, we used mean and standard deviation (SD) displays, and for categorical variables using number (*n*) and percentage (%) displays. According to the presence of LBP, we divided the participants into the non-LBP group and the LBP group. We used *t*-test to compare whether differences between non-LBP group and LBP group were significant for continuous variables, and the chi-square test was used to compare differences between non-LBP group and LBP group in categorical variables. We used multiple logistic regression models to assess the correlation between IR surrogates and LBP expressing the association with OR values and 95% confidence intervals (95% CI). Three models were constructed, in Model 1, no adjustment was made; Model 2 adjusted for the age, sex, ethnicity, marital status, PIR, and education level; Model 3 adjusted for the age, sex, ethnicity, marital status, PIR, education level, hypertension, smoke, alcohol user, fasting total cholesterol, HDL cholesterol, LDL cholesterol. *p*-value <0.05 was considered to be statistically significant.

## 3 Results

**Figure 1:**
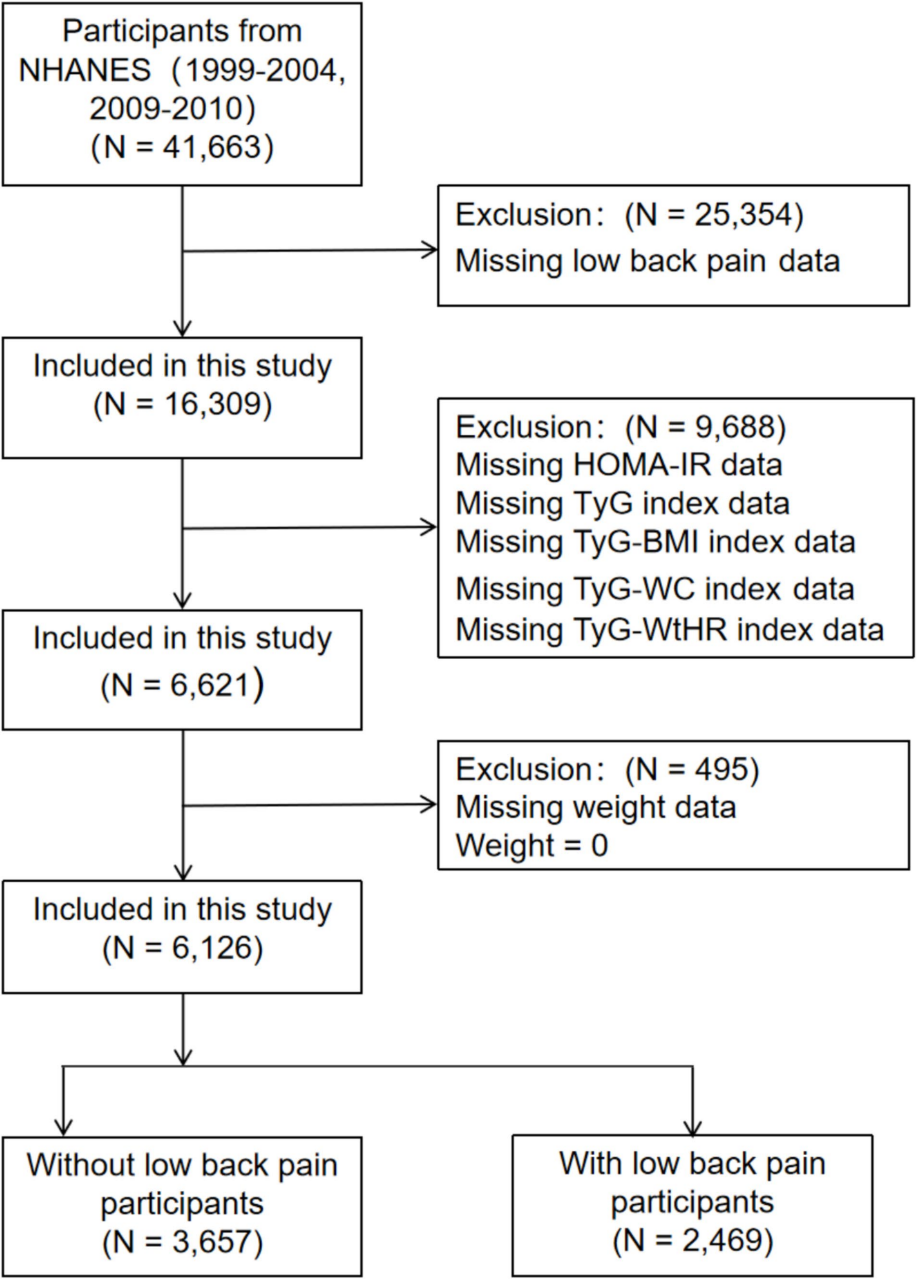
Flowchart of the study. HOMA-IR, homeostatic model assessment of insulin resistance; TyG, triglyceride glucose; TyG-BMI, triglyceride glucose with body mass index; TyG-WC, triglyceride glucose with waist circumference; TyG-WtHR, triglyceride glucose with the ratio of waist circumference divided by height.

From NHANES (1999–2000, 2001–2002, 2003–2004, and 2009–2010) obtained 41,663 participants, 25,354 participants missing LBP data were excluded. Again excluding 9,688 participants missing the data of HOMA-IR, TyG, TyG-BMI, TyG-WC, and TyG-WtHR. Excluding 495 participants lacking weight information or the value of weight is zero. Finally, 6,126 participants were included in this study. The selection flowchart of subjects is presented in Figure 1.

### 3.1 Characteristics of the study participants

Table 1 displays the weighted baseline characteristics of the 6,126 participants including 3,657 non-LBP participants and 2,469 LBP participants. The distribution of age and gender among the participants did not show any significant differences. Non-Hispanic White (74.88%), >3.5 PIR (38.49%), college graduate or above (48.68%), overweight or obese (68.23%) participants accounted for a higher portion among LBP group.

**Table 1:** Weighted baseline characteristics of participants in the groups of non-LBP and LBP.

Variable	Non-LBP group (*N* = 3,657)	LBP group (*N* = 2,469)	*p*-value
Age	45.26 (0.48)	45.84 (0.46)	0.22
Sex			0.11
Female	1,853 (50.60)	1,352 (53.25)	
Male	1,804 (49.40)	1,117 (46.75)	
Ethnicity			0.01
Non-Hispanic White	1797 (70.64)	1,356 (74.88)	
Non-Hispanic Black	662 (11.21)	387 (9.27)	
Mexican American	923 (7.98)	511 (6.59)	
Other	275 (10.18)	215 (9.25)	
Marital status			0.07
Married or living with partner	2,263 (65.32)	1,543 (68.25)	
Other	1,285 (34.68)	842 (31.75)	
PIR			<0.0001
0–1.3 PIR	847 (17.44)	703 (24.05)	
>1.3–3.5 PIR	1,309 (36.11)	869 (37.46)	
>3.5 PIR	1,196 (46.44)	692 (38.49)	
Education level			<0.0001
Less than 9th grade	552 (6.36)	373 (7.93)	
High school grade or equivalent	1,358 (35.33)	1,033 (43.39)	
College graduate or above	1741 (58.31)	1,060 (48.68)	
Smoke			<0.0001
Never	1967 (52.96)	1,125 (43.76)	
Former	979 (25.95)	681 (26.60)	
Now	708 (21.09)	661 (29.64)	
Alcohol use			0.001
Never	523 (11.95)	279 (10.48)	
Former	696 (16.61)	495 (18.70)	
Mild	1,159 (36.75)	744 (31.85)	
Moderate	457 (15.79)	343 (16.21)	
Heavy	651 (18.90)	502 (22.76)	
Hypertension			<0.001
No	2,249 (67.45)	1,406 (62.09)	
Yes	1,406 (32.55)	1,062 (37.91)	
Diabetes			0.002
No	3,206 (94.03)	2,112 (91.74)	
Yes	281 (5.97)	221 (8.26)	
BMI			<0.001
Underweight	51 (1.72)	37 (2.01)	
Normal	1,197 (35.28)	660 (29.76)	
Overweight	1,322 (34.73)	882 (34.09)	
Obese	1,087 (28.27)	890 (34.14)	
HOMA-IR	2.86 (0.06)	3.36 (0.10)	<0.0001
TyG	8.67 (0.02)	8.73 (0.01)	0.01
TyG-BMI	241.23 (1.62)	251.80 (1.69)	<0.0001
TyG-WC	830.42 (4.83)	860.69 (4.29)	<0.0001
TyG-WtHR	4.90 (0.03)	5.09 (0.02)	<0.0001

Continuous variables are presented as means with standard deviations (SD), while categorical variables are expressed as counts (*n*) and percentages (%). PIR, poverty income ratio; BMI, body mass index; HOMA-IR, homeostatic model assessment of IR; TyG, triglyceride glucose; TyG-BMI, triglyceride glucose with body mass index; TyG-WC, triglyceride glucose with waist circumference; TyG-WtHR, triglyceride glucose with the ratio of waist circumference divided by height.

### 3.2 Associations between IR surrogates and LBP

All of these five indices showed significant association with LBP in the Model 3, after full adjustment for all covariates, HOMA-IR [OR = 1.052, 95% CI (1.018, 1.087), *p* = 0.003], TyG [OR = 1.431, 95% CI (1.013, 2.021), *p* = 0.042], TyG-BMI [OR = 1.003, 95% CI (1.002, 1.005), *p* < 0.0001], TyG-WC [OR = 1.001, 95% CI (1.001, 1.002), *p* < 0.0001], TyG-WtHR [OR = 1.268, 95% CI (1.155, 1.393), *p* < 0.0001]. Furthermore, we discretized the five IR indices that were originally continuous variables into quartiles for a sensitivity analysis. Compared with quartile 1 (Q1), quartile 4 (Q4) was 46.8% higher (*p* = 0.002) in HOMA-IR, Q4 was 61.1% higher (*p* < 0.001) in TyG-BMI, Q4 was 91.4% higher (*p* < 0.001) in TyG-WC. Q4 was 67.0% higher than Q1 of the TyG-WtHR index (*p* < 0.001). Furthermore, the *p* for trend indicated the statistically significant nature of the upward trend observed for HOMA-IR, TyG-BMI, TyG-WC, and TyG-WtHR in the fully adjusted model, implying that LBP risk increases with increasing degree of IR. Table 2 provides the detailed results.

**Table 2:** The results of logistic regression analysis on the association between insulin resistance surrogates and LBP.

Character	Model 1		Model 2		Model 3	
	OR (95% CI)	*p*	OR (95% CI)	*p*	OR (95% CI)	*p*
HOMA-IR	1.050 (1.027, 1.074)	<0.0001	1.045 (1.020, 1.071)	<0.001	1.052 (1.018, 1.087)	0.003
Q1	ref		ref		ref	
Q2	1.122 (0.968, 1.300)	0.124	1.036 (0.872, 1.232)	0.679	1.109 (0.921, 1.335)	0.267
Q3	1.144 (0.967, 1.354)	0.115	1.070 (0.890, 1.287)	0.465	1.118 (0.895, 1.397)	0.316
Q4	1.438 (1.218, 1.698)	<0.0001	1.334 (1.110, 1.603)	0.003	1.468 (1.160, 1.859)	0.002
*p* for trend		<0.0001		0.003		0.005
TyG	1.150 (1.032, 1.281)	0.012	1.064 (0.942, 1.201)	0.311	1.431 (1.013, 2.021)	0.042
Q1	ref		ref		ref	
Q2	1.088 (0.916, 1.292)	0.330	1.068 (0.893, 1.276)	0.464	1.079 (0.886, 1.316)	0.439
Q3	1.284 (1.093, 1.507)	0.003	1.197 (0.986, 1.452)	0.068	1.297 (0.962, 1.749)	0.086
Q4	1.336 (1.085, 1.645)	0.007	1.171 (0.933, 1.471)	0.169	1.494 (0.925, 2.414)	0.098
*p* for trend		0.003		0.107		0.077
TyG-BMI	1.003 (1.002, 1.004)	<0.0001	1.002 (1.001, 1.003)	<0.0001	1.003 (1.002, 1.005)	<0.0001
Q1	ref		ref		ref	
Q2	1.127 (0.937, 1.354)	0.199	1.108 (0.913, 1.344)	0.291	1.138 (0.918, 1.411)	0.229
Q3	1.193 (0.983, 1.448)	0.074	1.108 (0.902, 1.360)	0.322	1.185 (0.896, 1.568)	0.227
Q4	1.511 (1.263, 1.807)	<0.0001	1.451 (1.200, 1.754)	<0.001	1.611 (1.235, 2.101)	<0.001
*p* for trend		<0.0001		<0.001		0.001
TyG-WC	1.001 (1.001, 1.001)	<0.0001	1.001 (1.000, 1.001)	<0.0001	1.001 (1.001, 1.002)	<0.0001
Q1	ref		ref		ref	
Q2	1.276 (1.051, 1.548)	0.014	1.250 (1.012, 1.543)		1.323 (1.047, 1.673)	
Q3	1.246 (1.033, 1.502)	0.022	1.157 (0.944, 1.418)		1.257 (0.970, 1.628)	
Q4	1.648 (1.334, 2.037)	<0.0001	1.565 (1.249, 1.961)		1.914 (1.388, 2.639)	
*p* for trend		<0.0001		<0.001		<0.001
TyG-WtHR	1.205 (1.130, 1.285)	<0.0001	1.171 (1.091, 1.256)	<0.0001	1.268 (1.155, 1.393)	<0.0001
Q1	ref		ref		ref	
Q2	1.064 (0.871, 1.298)	0.538	1.045 (0.852, 1.281)	0.670	1.085 (0.870, 1.354)	0.457
Q3	1.324 (1.123, 1.562)	0.001	1.251 (1.041, 1.504)	0.018	1.340 (1.046, 1.718)	0.022
Q4	1.536 (1.247, 1.892)	<0.001	1.428 (1.140, 1.788)	0.003	1.670 (1.257, 2.220)	<0.001
*p* for trend		<0.0001		<0.001		<0.001

Model 1: No adjustment was made for any covariate. Model 2: Adjusted by age, sex, ethnicity, marital status, poverty income ratio, and education level. Model 3: Adjusted by age, sex, ethnicity, marital status, poverty income ratio, education level, hypertension, smoking, alcohol use, fast total cholesterol, HDL cholesterol, and HDL cholesterol.

### 3.3 Subgroup analysis

**Figure 2:**
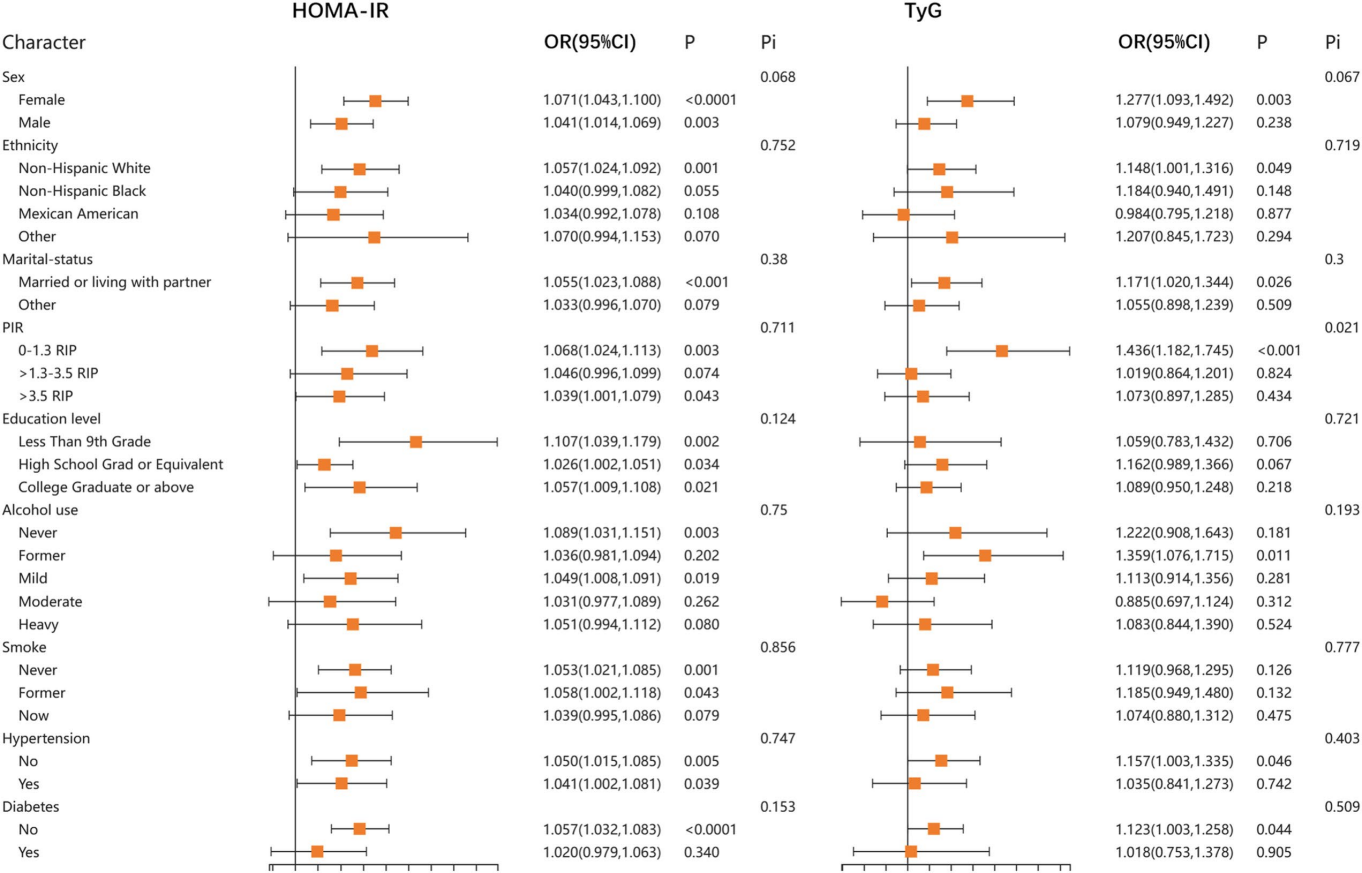
Stratified associations between HOMA-IR, TyG, and LBP according to baseline characteristics. HOMA-IR, homeostatic model assessment of insulin resistance; TyG, triglyceride glucose index; PIR, poverty income ratio; Pi, P for interaction.

**Figure 3:**
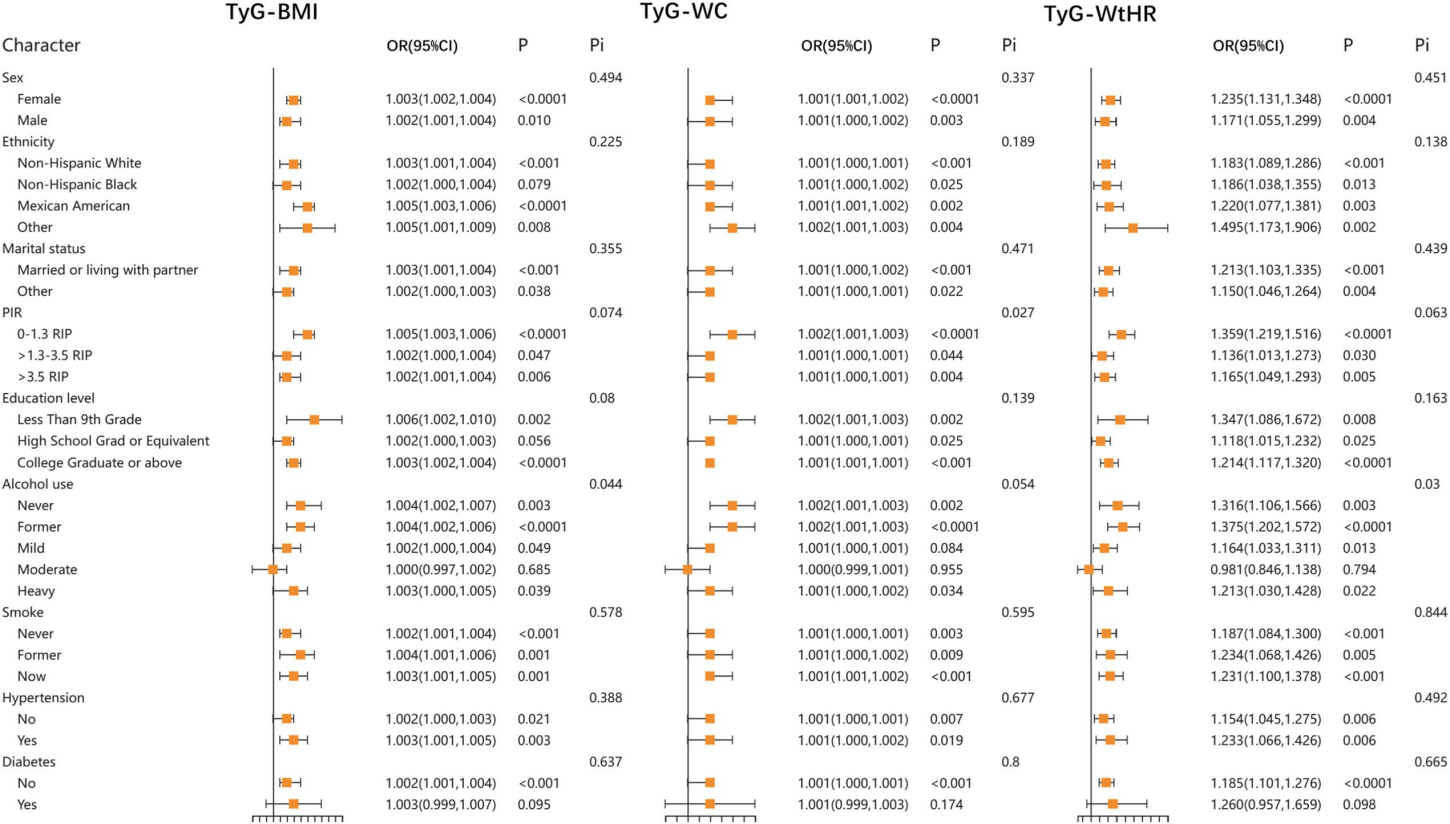
Stratified associations between TyG-BMI, TyG-WC, TyG-WtHR, and LBP according to baseline characteristics. TyG-BMI, triglyceride glucose with body mass index; TyG-WC, triglyceride glucose with waist circumference; TyG-WtHR, triglyceride glucose with the ratio of waist circumference divided by height; PIR, poverty income ratio; Pi, P for interaction.

In addition, to further confirm the stability of the results, we performed stratified analyses for HOMA-IR, TyG, TyG-BMI, TyG-WC, and TyG-WtHR. The results demonstrated that the relationship between HOMA-IR, TyG, TyG-BMI, TyG-WC, and TyG-WtHR index and LBP was stable in most stratified populations (*p*-interaction >0.05). Detailed results of the stratified analysis are presented in Figures 2, 3. Moreover, the results of the logistic regression analysis stratified by gender are presented in Table 3. TyG did not show a significant correlation with LBP in the male population.

**Table 3:** The results of logistic regression analysis on the association between IR surrogates and LBP stratified by gender.

Characters	Sex	Number	Model 1		Model 2		Model 3	
			OR (95% CI)	*p*	OR (95% CI)	*p*	OR (95% CI)	*p*
HOMA-IR	Female	3,205	1.071 (1.043, 1.100)	<0.0001	1.063 (1.033, 1.093)	<0.0001	1.073 (1.037, 1.111)	<0.001
	Male	2,921	1.041 (1.014, 1.069)	0.003	1.034 (1.007, 1.062)	0.016	1.038 (0.996, 1.082)	0.077
TyG	Female	3,205	1.277 (1.093, 1.492)	0.003	1.145 (0.947, 1.385)	0.157	1.322 (0.845, 2.070)	0.215
	Male	2,921	1.079 (0.949, 1.227)	0.238	1.005 (0.876, 1.154)	0.937	1.553 (0.906, 2.663)	0.106
TyG-BMI	Female	3,205	1.003 (1.002, 1.004)	<0.0001	1.003 (1.002, 1.004)	<0.0001	1.003 (1.001, 1.005)	<0.001
	Male	2,921	1.002 (1.001, 1.004)	0.010	1.002 (1.000, 1.003)	0.070	1.003 (1.001, 1.005)	0.008
TyG-WC	Female	3,205	1.001 (1.001, 1.002)	<0.0001	1.001 (1.001, 1.002)	<0.001	1.002 (1.001, 1.002)	<0.001
	Male	2,921	1.001 (1.000, 1.002)	0.003	1.001 (1.000, 1.001)	0.042	1.001 (1.000, 1.002)	0.004
TyG-WtHR	Female	3,205	1.235 (1.131, 1.348)	<0.0001	1.205 (1.089, 1.334)	<0.001	1.258 (1.086, 1.456)	0.003
	Male	2,921	1.171 (1.055, 1.299)	0.004	1.114 (0.996, 1.246)	0.059	1.250 (1.077, 1.450)	0.004

Model 1: No adjustment was made for any covariate. Model 2: Adjusted by age, ethnicity, marital status, poverty income ratio, and education level. Model 3: Adjusted by age, ethnicity, marital status, poverty income ratio, education level, hypertension, smoking, alcohol use, fast total cholesterol, HDL cholesterol, and LDL cholesterol. The red color represents a significant *p*-value (*p*<0.05).

## 4 Discussion

In this study, data from four NHANES (1999–2000, 2001–2002, 2003–2004, and 2009–2010) cycles were utilized to assess the associations between HOMA-IR, TyG, TyG-BMI, TyG-WC, TyG-WtHR, and LBP. After adjusting for all the covariates, there was still a stable positive correlation between HOMA-IR, TyG, TyG-BMI, TyG-WC, TyG-WtHR, and LBP. Further stratified analysis also indicated that these results were stable in most of the subgroups. To the best of our knowledge, this is the first study using NHANES data to investigate the relationship between IR and LBP.

Currently, the internationally accepted gold standard for evaluating IR is hyperinsulinemic euglycemic clamp (HEC) (21). The principle is that glucose and insulin are infused simultaneously to maintain blood glucose levels within the range of 4.4 to 5.0 mmol/L. During this state, the infusion rate of exogenous glucose matches the peripheral tissue glucose utilization rate. IR severity is assessed by quantifying the rate of insulin-mediated glucose metabolism (22). Although the measurement results of this method are stable and reproducible, however, its widespread adoption in clinical practice is hindered by its considerable technical complexity, lengthy duration, and substantial cost implications (23). To find a simple, practical and reliable tool to assess body insulin sensitivity, HOMA-IR based on fasting insulin (FINS) and fasting blood glucose (FPG) levels has emerged (18). The index can be calculated only by obtaining FINS and FPG. It has the characteristics of simple operation, cheap price and almost no damage to patients, so it is widely used in practice. However, the determination of FINS is not a routine laboratory test in clinical practice, so the triglyceride-glucose (TyG) index, calculated by fasting triglycerides and fasting glucose level, was also developed (19). A study (24) showed that TyG links IR even more closely than HOMA-IR with IR. In addition, some other IR substitution indices deriving TyG, such as TyG-BMI, TyG-WC, and TyG-WtHR, also show a closer relationship with IR than HOMA-IR, and even have a stronger ability to predict IR or IR-related diseases than TyG (25, 26).

The results of this study revealed that HOMA-IR, TyG, TyG-BMI, TyG-WC, and TyG-WtHR index are all associated with higher risk of LBP and that TyG-BMI, TyG-WC, and TyG-WtHR index have even more stable associations with LBP than TyG alone. Our conclusions are consistent with the previous studies to some extent. Cross-sectional studies from Japan noted a significant association between LBP and metabolic syndrome, however, there were significant gender differences in this relationship, with a significantly higher prevalence of metabolic syndrome in women with non-LBP, but the relationship was not significant in the male group (27, 28). Another study reached the same conclusion that patients with LBP had a higher prevalence of metabolic syndrome (29). This is consistent with our study, although most IR indicators showed significant association with LBP, the correlation was more stable in the female population than male population, especially TyG. In addition, their study also found that LBP patients with metabolic syndrome have higher BMI and waist circumference relative to LBP patients without metabolic syndrome. These results are partly in agreement with the present study, where the index of TyG combining various obesity-related indices showed a more stable correlation with LBP than the TyG index alone.

Despite the etiology of LBP is complex and varied, intervertebral disc degeneration is one of the main contributing causes of LBP, accounting for about 26%–42% of patients with LBP (30). A previous Mendelian randomization analysis from our team found that triglycerides was able to mediate T2D to promote intervertebral disc degeneration (31). It is already an established fact that obesity is a risk factor for Intervertebral disc degeneration (32, 33). Obesity can also develop into IR and chronic low-grade systemic inflammation through lipotoxicity, promoting the development of LBP (34). Similar conclusions have been found in several previous studies showing that TyG-BMI, TyG-WC, and TyG-WtHR are more robust in their associations with IR or IR-related diseases than TyG alone. A cross-sectional study (20) using NHANES data found that TyG-WC had a better ability to identify IR than TyG alone. In addition, the research of Dang et al. (25) showed that TyG-WC and TyG-WtHR have a higher accuracy in cardiovascular disease mortality prediction compared to TyG and TyG-BMI, and that is expected to be a more effective indicator for identifying patients at early risk of cardiovascular disease. The higher predictive power of TyG-BMI, TyG-WC, and TyG-WTHR for LBP may be attributed to the following reasons. First, these indicators combine triglyceride of fasting, fasting glucose and obesity-related indicators, able to consider the effect of both risk factors, IR and BMI, on LBP. Second, waist circumference and waist height ratio are the indicators of abdominal obesity, patients with abdominal obesity have a higher risk of IR than ordinary obese patients, therefore, TyG-WC and TyG-WtHR can demonstrate a more stable correlation with IR or IR-related diseases.

This study has some of the following advantages. First, we obtained widely representative large-scale survey data from NHANES, which improves the stability and generalizability of our results. Second, we comprehensively evaluated the association of IR with LBP using five IR surrogates. Finally, stratified analyses were performed to assess the stability of the results. However, we also have some inevitable shortcomings. First, the LBP data used for analysis were derived from retrospective questionnaires that inevitably cause recall bias. Second, the design of this study was a cross-sectional study, which prevented us from further exploring the causal relationship between LBP and IR. Prospective studies are needed to establish a causal relationship and to determine whether improving IR can reduce LBP incidence or severity. Finally, we have to acknowledge that NHANES data primarily represent the US population, and its generalizability to non-US populations may be limited. Further validation in other populations are needed.

## 5 Conclusion

IR is associated with an increased risk of LBP. Compared to TyG alone, TyG-WC, TyG-BMI and TyG-WtHR showed a more stable correlation with LBP. Future research should explore whether targeting IR through lifestyle modifications, pharmacological interventions, or combined approaches could help alleviate LBP symptoms.

## Data Availability

The original contributions presented in the study are included in the article/supplementary material, further inquiries can be directed to the corresponding authors.

## Footnotes

^1^ https://wwwn.cdc.gov/nchs/nhanes/
